# Two new species of *Mylochromis* (Cichlidae) from Lake Malawi, Africa

**DOI:** 10.1111/jfb.16014

**Published:** 2024-12-10

**Authors:** George F. Turner

**Affiliations:** ^1^ School of Environmental & Natural Sciences, Bangor University Bangor UK; ^2^ Vertebrates Division Natural History Museum London UK

**Keywords:** African cichlid, molariform, species description, taxonomy

## Abstract

Two new species of *Mylochromis* Regan 1920 are described from specimens collected on shallow rocky habitats on the northwestern coast of Lake Malawi. The generic designation is based on their prominent oblique‐striped pattern and lack of any of the unique features of other Malawi cichlid genera with this pattern. *Mylochromis rotundus* sp. nov. is distinguished from most congeneric species by its relatively deep, rounded body and lack of enlarged pharyngeal teeth. It is further distinguished from *Mylochromis semipalatus* and *Mylochromis melanonotus* (if they are different species) by its relatively shorter snout. *Mylochromis durophagus* sp. nov. is distinguished from most congeneric species by its strongly molarized pharyngeal dentition. It differs from *Mylochromis mola* by having a shorter snout, a less acutely pointed head profile, a deeper body, and a more continuous oblique stripe. *M. durophagus* has a much less steep head profile than *Mylochromis anaphyrmus* or *Mylochromis sphaerodon*. It is suggested that *M. rotundus* was previously identified informally as *Mylochromis* sp. “mollis north,” and *M. durophagus* as *Mylochromis* sp. “mollis chitande.” Lectotypes are designated for comparator species *M. sphaerodon* and *Mylochromis subocularis*.

## 1 INTRODUCTION

Lake Malawi's cichlid fish fauna presents a challenge to researchers (Snoeks, 2004) as a result of the exceptional number of species (around 800: Konings, 2016), many of which are extremely similar morphologically (Turner et al., 2001). The rate of species description is slow, and many species, even some well‐known ones, remain undescribed, rendering them ineligible to receive IUCN red listing, or incorporation into standard reference systems such as FishBase and GBIF.

Most Malawian cichlids are haplochromines, closely related to the riverine *Astatotilapia calliptera* (Günther, 1894) (Eccles & Trewavas, 1989; Joyce et al., 2011; Malinsky et al., 2018). Among Lake Malawi haplochromines, a number of species show an oblique stripe along the flanks, from the nape to the caudal peduncle or caudal fin; this pattern is believed to be unique among haplochromine cichlids (Eccles & Trewavas, 1989). In the last major revision of a large section of the Malawian haplochromines, Eccles and Trewavas (1989) assigned 22 of the oblique‐striped species to 10 genera: *Aristochromis* Trewavas, 1935, *Buccochromis* Eccles & Trewavas, 1989, *Caprichromis* Eccles & Trewavas, 1989, *Champsochromis* Boulenger, 1915, *Corematodus* Boulenger, 1897, *Docimodus* Boulenger, 1897, *Lichnochromis* Trewavas, 1935, *Platygnathochromis* Eccles & Trewavas, 1989, *Taeniolethrinops* Eccles & Trewavas, 1989, and *Tramitichromis* Eccles & Trewavas, 1989, largely on the basis of specialized head and jaw morphology; in some cases, these genera also included species with different melanin patterns (*Corematodus, Tramitichromis*). The remaining 16 species were placed in the genus *Maravichromis* Eccles & Trewavas, 1989, type *Haplochromis ericotaenia* Regan, 1922, but they were unable to identify any synapomorphic characters for the genus, and its definition was based on the shared possession of the oblique‐striped pattern and the lack of putatively apomorphic traits used to define the other genera incorporating oblique‐striped species. *Maravichromis* was later found to be a subjective junior synonym of *Mylochromis* Regan, 1920 (Derijst & Snoeks, 1992). The genus was originated in a footnote to Regan's paper on Tanganyikan cichlids, with the type species *Chromis lateristriga* Günther, 1864, but in his paper on Malawian cichlids published in 1922, that species was included in the genus *Haplochromis* Hilgendorf, 1888, and *Mylochromis* was not listed in the synonymy. No subsequent revision of *Mylochromis* has taken place, and the definition for *Maravichromis* has been assumed to be rolled over to *Mylochromis*. Subsequently, however, Konings (1993a) revised the genus *Sciaenochromis* Eccles & Trewavas, 1989, expelling two species to *Mylochromis*, then proposing that *Mylochromis semipalatus* (Trewavas, 1935) should be considered a junior synonym of *Platygnathochromis melanonotus* (Regan, 1922), that *Platygnathochromis*, in turn, is a junior synonym of *Mylochromis* (Konings, 1993b), and also that *Chromis subocularis* Günther, 1894 be moved from *Placidochromis* to *Mylochromis* (Konings, 1995). Turner and Howarth (2001) described two additional species. However, the synonymy of *M. semipalatus* with *Mylochromis melanonotus* was rejected by Snoeks and Hanssens (2004) although maintained by Konings (2016). Fricke et al. (2024) currently accept the validity of *M. semipalatus*, bringing the total number of species to 22. Snoeks and Hanssens (2004) suggested the genus was in desperate need of revision, that additional undescribed species were intermediate between this genus and *Sciaenochromis* and *Stigmatochromis* Eccles & Trewavas, 1989, and that in addition to the three species then recognized to have heavily molariform pharyngeal dentition, they tentatively identified an additional 11 morphological groups. Many probably undescribed species are illustrated in underwater photographs by Konings (2016), but in most cases, there are no specimens available to examine. Clearly, much further work is required.

During the examination of a collection of material supporting a programme of genome sequencing (see Malinsky et al., 2018; Svardal et al., 2019; Turner et al., 2022 for preliminary results), two new species were identified that fall within the current definition of the genus *Mylochromis* and are formally described in the present work.

## 2 METHODS

Specimens of the new species examined were in existing museum collections at Cambridge University. Counts and measurements were carried out following the methods of Snoeks (2004), using digital calipers and a low‐power magnifying desk lamp, with additional examination using a binocular dissecting microscope. Angles were measured from photographs using an online protractor (ginifab.com/feeds/angle_measurement/).

Additional material: ***Mylochromis anaphyrmus*** (Burgess & Axelrod, 1973) BMNH 2024.6.25.1–4: 4 specimens 121.3–130.7 mm standard length (SL), trawled from 60 m depth at Chilinda, southeast arm of Lake Malawi (−13.743, 35.053), collected by G. Turner 1991; ***Mylochromis chekopae*** Turner & Howarth, 2001: BMNH 1996.10.14.98, holotype, male, trawled from 20 m depth off Chekopa on the eastern shore, southeast arm, Lake Malawi (13° 53′ S, 35° 07′ E), Turner, November 19, 1991; BMNH 1996.10.14.99–120, paratypes, 22 specimens, 103.0–122.4 mm SL, collection data as holotype; ***Mylochromis ericotaenia*** (Regan, 1922): BMNH 1921.9.6.148–149 (1) lectotype of *H. ericotaenia*, 57.9 mm SL, collected from Lake Nyasa by Wood 1920; BMNH 1935.6.14.2412–2414, 4 specimens, 63.4–81.3 mm SL, collected from Lake Nyasa by Christy 1925; BMNH 1935.6.14:2381–2385, six mature males 116.1–162.7 mm SL, collected from Deep Bay (Chilumba), Malawi, by Christy 1925; BMNH 1935.6.14.2401–2402, two apparent mature males, 131.9–137.9 mm SL collected from Deep Bay by Christy 1925; ***Mylochromis labidodon*** (Trewavas, 1935): BMNH 1935.6.14.2417, lectotype of *Haplochromis labidodon*, 100 mm SL, collected from Mwaya, Lake Nyasa, Tanzania by Christy 1925; BMNH 1935.6.14.2418–9, paralectotypes: three specimens: one dissected, others 84.9, 85.4 (both very soft and faded), collected with lectotype; BMNH 1935.6.14.2416, paralectotype, one specimen 149.9 mm SL from Deep Bay (Chilumba), Malawi, by Christy 1925; ***Mylochromis mola*** (Trewavas, 1935) three specimens: BMNH 1935.6.14.2357, lectotype of *Haplochromis mola*, 139.0 mm SL, collected from Vua, Lake Malawi, by Christy 1925; BMNH 1935.6.14.2358–2359, paralectotypes 110.2–122.4 mm SL, collected with lectotype; UMZC 2016.38.43 (field ID: D10‐J02), one specimen 89.2 mm SL, collected from Chiofu Bay (−13.533, 34.866), SCUBA, February 26, 2016; ***Mylochromis mollis*** (Trewavas, 1935) two specimens: BMNH 1935.6.14.1334, lectotype of *Haplochromis mollis*, 131.9 mm SL, collected from Monkey Bay by Christy 1925; BMNH 1935.6.14.1335: paralectotype 85.0 mm SL, collected with lectotype; ***Mylochromis obtusus*** (Trewavas, 1935), BMNH 1935.6.14.1453, holotype of *Haplochromis obtusus*, 190 mm SL, collected from southeast arm between Bar and Nkhudzi by Christy 1925; ***Mylochromis sphaerodon*** (Regan, 1922) two specimens: BMNH 1921.9.6.146, lectotype (here designated) of *Haplochromis sphaerodon*: figured by E. Fasken in Eccles & Trewavas, 1989, 90.2 mm SL, collected from “Lake Nyasa” by Wood; paralectotype 90.9 mm SL, collected with lectotype; ***Mylochromis subocularis*** (Günther, 1894): BMNH 1893.11.15.33, lectotype of *C. subocularis* (here designated), figured by Günther, 1894 (drawing) and Eccles & Trewavas, 1989 (photograph): 94.8 mm SL, collected from Lake Nyasa and Upper Shire River, presented by H.H. Johnston; BMNH 1935.6.14.1180–1189, 19 specimens 82.3–130.4 mm SL, collected by Christy, Bar House, Lake Malawi; BMNH 1966.7.25.15. 1 adult male, 113.5 mm SL, collected from Liwonde, Upper Shire River below barrage, by Malawi Fisheries Research Unit/E. Trewavas. UMZC 2021.42.7 (field ID: D23‐D03) adult male, not measured, collected from Chirimila catch, Thumbi West Island, Malawi, January 29, 2017; UMZC uncataloged (field code: D12‐E09), adult male, not measured (photo only), trawled at 20 m depth off Makanjila (−13.769, 34.985), March 2, 2016. Information on other species was obtained from uncataloged material and from existing literature, mainly Eccles and Trewavas (1989).

## 3 RESULTS

### 3.1 *Mylochromis rotundus* new species

urn:lsid:zoobank.org:act:54696C3B‐768C‐4405‐BDA6‐9777A3F3A57D.

**FIGURE 1:**
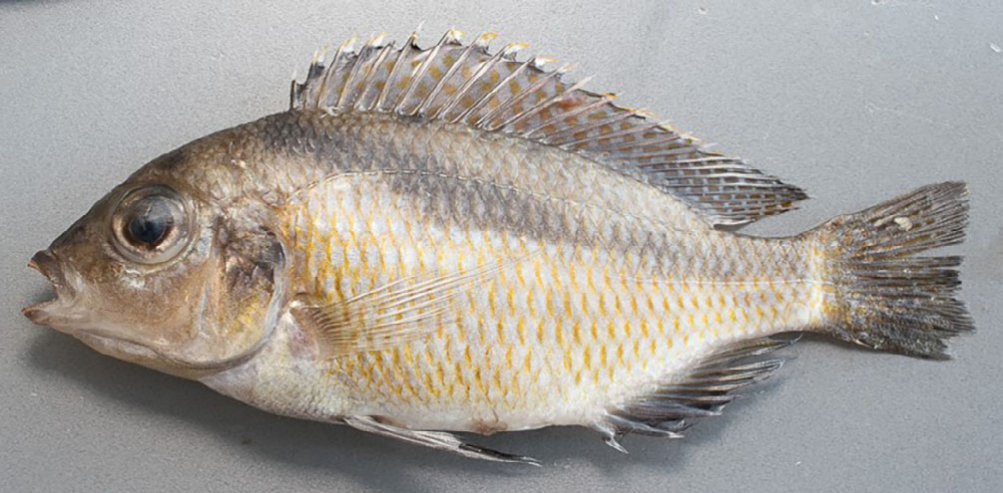
*Mylochromis rotundus*, holotype, UMZC 2016.25.9, 99.7 mm standard length (SL) male, freshly collected.

**FIGURE 2:**
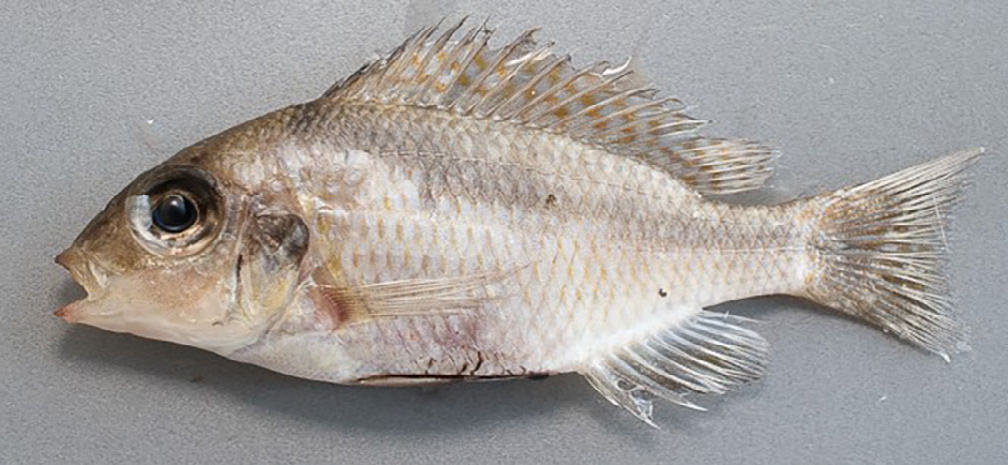
*Mylochromis rotundus*, paratype, UMZC 2016.25.10, 71.5 mm standard length (SL) female, freshly collected.

**FIGURE 3:**
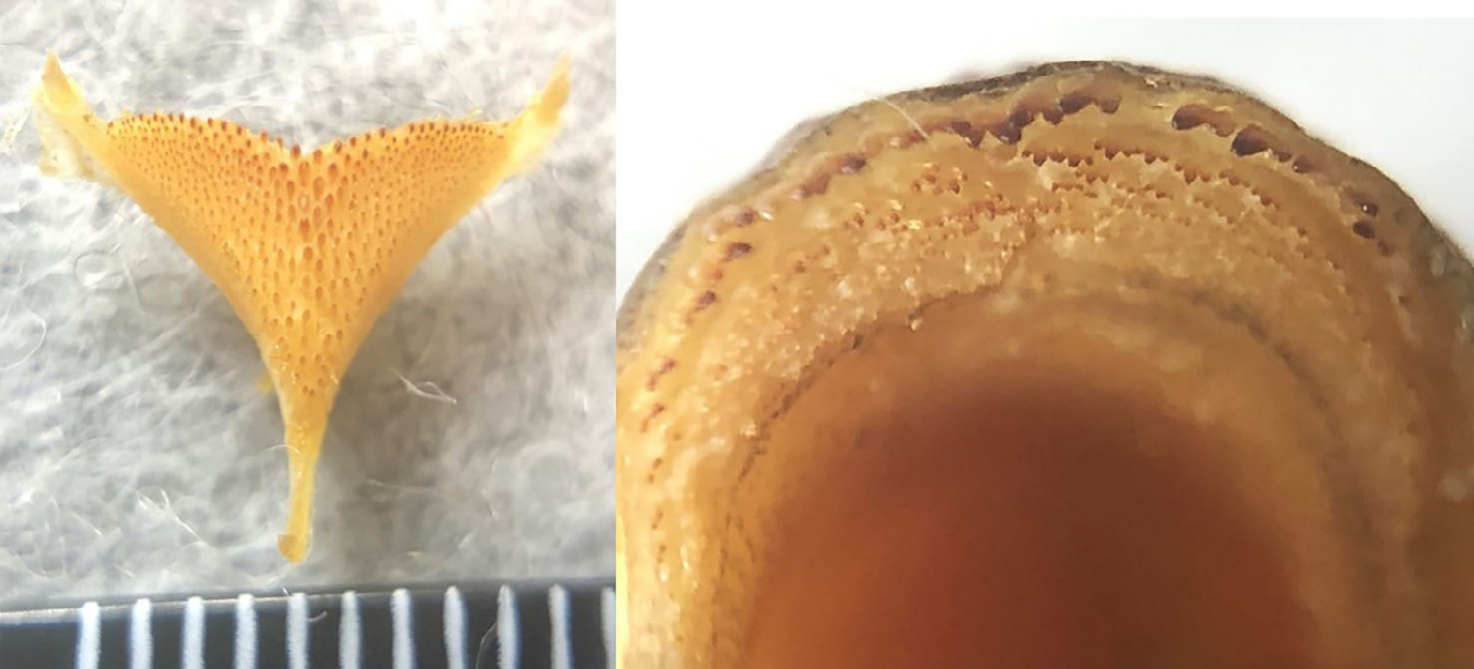
*Mylochromis rotundus*, holotype, lower pharyngeal (left) and oral dentition (lower jaw, right).

Holotype: University Museum of Zoology, Cambridge: UMZC 2016.25.9 apparent male, 99.7 mm SL, collected by scuba at Mphanga Rocks February 23, 2016, by Malawi Cichlid Genomic Diversity Survey (MCGDS) (Figures 1, 2, 3).

Paratypes (four): three apparent males: UMZC 2016.5.4, 88 mm SL; 2016.5.5 (lower jaw damaged), UMZC 104.8 mm SL; UMZC 2016.5.11, 93.5 mm SL and one female UMZC 2016.25.10, 71.5 mm SL collected with holotype.

Etymology: “rotundus” = round, referring to the rounded body shape and short, rounded snout in particular.

Diagnosis: the lower jaw dentition is “*Placidochromis*‐type,” with the outer series extending relatively straight toward the posterior end of the jaw. The rounded body, short snout, and large eye enable the species to be readily distinguished from most other known oblique‐striped species. Body depth of >38% SL and the lack of enlarged pharyngeal teeth distinguish the species from all other known congeneric species apart from *M. semipalatus* and *M. melanonotus* (data from Eccles & Trewavas, 1989). These two are considered conspecific by Konings (1993b). The latter has a distinctively flattened lower jaw at the symphysis. Both of these have relatively much longer snouts than *M. rotundus* (see Figure 9). In *M. melanonotus*: snout length 1.5 to almost 2× eye diameter (Eccles & Trewavas, 1989), *M. semipalatus* snout 1.5× eye diameter (Eccles & Trewavas, 1989) versus 0.9–1.2× in *M. rotundus*.

Description: body measurements and counts are presented in Table 1. *M. rotundus* is a medium sized (< 105 mm SL), laterally compressed (maximum body depth 2.5–2.6 times maximum width) cichlid fish with a rounded head profile, terminal mouth, and large eyes (31.8%–38.8% head length [HL]) (Figures 1 and 2). When a melanin pattern is visible, it is dominated by a broad continuous oblique stripe.

**TABLE 1:** Morphometrics and meristics of *Mylochromis rotundus* sp. nov., holotype and four paratypes.

	Holotype	Paratypes: mean	Minimum	Maximum
Standard length (SL, mm)	99.7	89.5	71.5	104.8
As % SL				
Body depth	40.7	39.8	38.6	40.4
Head length	32.2	32.9	31.9	35.0
Dorsal‐fin base length	56.5	55.9	53.4	57.6
Anal‐fin base length	19.1	18.7	18.3	19.1
Predorsal length	37.2	38.1	36.8	40.4
Pre‐anal length	71.2	69.9	68.7	71.8
Prepelvic length	34.8	34.2	32.1	36.5
Preventral length	42.5	40.6	38.8	42.0
Caudal peduncle length	14.1	14.8	13.6	15.9
Caudal peduncle depth	13.1	12.4	11.5	13.4
As % head length				
Head width	49.2	48.6	44.4	51.0
Interorbital width	29.6	26.9	22.8	28.8
Snout length	37.7	36.0	34.8	37.4
Lower jaw length	35.2	33.8	30.6	38.0
Premaxillary pedicel length	28.0	26.6	25.2	27.8
Cheek depth	23.1	17.0	16.3	17.6
Eye diameter	31.8	34.8	32.0	38.8
Lachrymal depth	26.2	23.9	21.2	25.3
Ratios				
Maximum depth/head width	2.57	2.49	2.46	2.54
Caudal peduncle length/depth	1.08	1.20	1.11	1.31
Meristics				
Upper gill rakers	3		4	5
Lower gill rakers	13		10	11
Dorsal‐fin spines	16		16	17
Dorsal‐fin rays	12		10	11
Anal‐fin rays	9		9	9
Longitudinal‐line scales	36		32	33
Cheek scales	4		3	3

All specimens are relatively deep bodied and laterally compressed, with deepest part of body generally around seventh dorsal‐fin spine. Anterior upper lateral profile convex, 40–45° to horizontal anteriorly, slightly concave above eye, continuing straight to tip of snout, with no obvious bulge made by premaxillary pedicel. Jaws isognathous to slightly retrognathous, jaw teeth prominent even when mouth closed. Tip of snout well above level of upper insertion of pectoral fin and about level with the bottom of eye. Lower profile curves gently from lower jaw tip to insertion of pelvic fins, then almost straight from pelvic to first anal spine. Mouth relatively small, gape angled about 32–45° to horizontal and lips relatively thin. Posterior end of maxilla well in front of anterior margin of eye. Eye large, circular, and generally below the head profile in lateral view. Lachrymal wider than deep with five openings.

Flank scales weakly ctenoid, with cteni becoming reduced dorsally, particularly anteriorly above upper lateral line, where they transition into a cycloid state. Scales on chest relatively large (e.g., in type, largest scale 2 mm vs. width across insertion of pelvics, 7.6 mm). Gradual transition in size from larger flank scales to smaller chest scales, typical in non‐mbuna Malawian endemic haplochromines (Eccles & Trewavas, 1989). Caudal fin densely scaled, over at least proximal three‐fourths.

Cephalic lateral‐line pores fairly inconspicuous, and flank lateral line shows usual cichlid pattern of separate upper and lower portions, with zero to four pored scales after kink in upper lateral line and two to three smaller pored scales after line of flexion of hypurals.

Pectoral fins relatively short, extending beyond vent but not to first anal spine, whereas pelvics occasionally just reach first anal spine base. Filaments of dorsal and anal fins reaching just past base of caudal fin. Caudal emarginate.

Lower jaw relatively sturdy and broad, with marked mental process. Outer series of teeth in lower jaw stout, erect, prominent, subequally bicuspid with two large rounded cusps, generally deeply implanted in fleshy gums (Figure 3). Upper jaw outer series similar, but in some specimens relatively long shafts visible. In both upper and lower jaws, three to five irregular inner series of relatively large, erect, stout tricuspid teeth with blunt tips.

Lower pharyngeal bone small, lightly built, Y‐shaped, carrying small, short, slender blunt teeth (Figure 3). None notably enlarged, although central teeth tend to be larger than lateral ones. Gill rakers widely spaced, pointed, finger‐like with thick bases.

**FIGURE 4:**
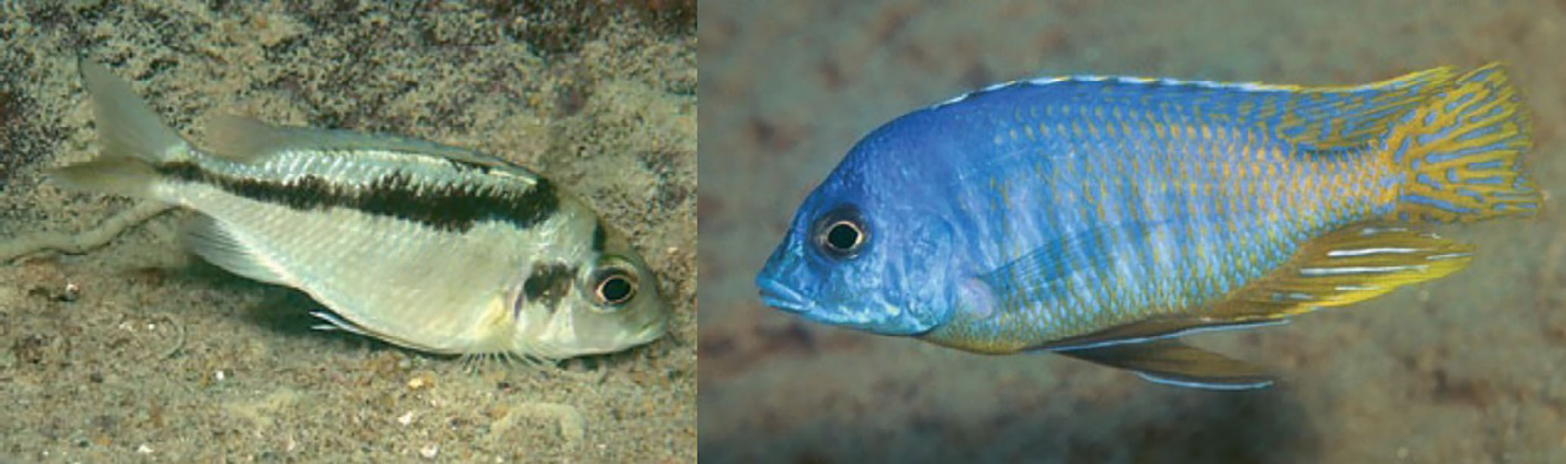
Live colouration of specimens provisionally identified as *Mylochromis* sp. “mollis north,” which may represent *Mylochromis rotundus*: female/immature from Katale Island, Chilumba (left), mature male from Hora Mhango, northwest coast between S. Rukuru River and Ruarwe (right). Photos A. Konings.

Preserved and fresh colouration of female beige, paler on chest and belly, flank scales with a brownish spot anteriorly (Figure 2). Strong dark oblique stripe from nape to the caudal‐fin base, extending into fin rays. Stripe is continuous but has a “stepped” form of thinner lines joining elongated midlateral and suprapectoral blotches. A large dark blotch on upper half of operculum. Dorsal fin with dull orange spots throughout, and caudal, anal, and pelvic fins dusky. Preserved and freshly collected mature males darker with golden spots on majority of flank scales (Figure 1). Dorsal, caudal, anal, and pelvic fins dark, dorsal and caudal with numerous orange spots. Dorsal‐fin lappets white with orange tips. It is possible that this is not the full breeding dress and that fully courting males are bright blue (Figure 4).

Distribution and ecology: known only from a collection from shallow water at Mphanga Rocks near Chilumba in the northwestern part of Lake Malawi. Photographs under the name *Mylochromis sp*. “mollis north,” which may or may not be of this species, are shown from the coast between Ruarwe and Chilumba (Konings, 2016; Figure 4). It appears to be a species of rocky habitats.

### 3.2 *Mylochromis durophagus* new species

urn:lsid:zoobank.org:pub:B29807EB‐60A6‐4389‐A235‐993B72E39C9B.

**FIGURE 5:**
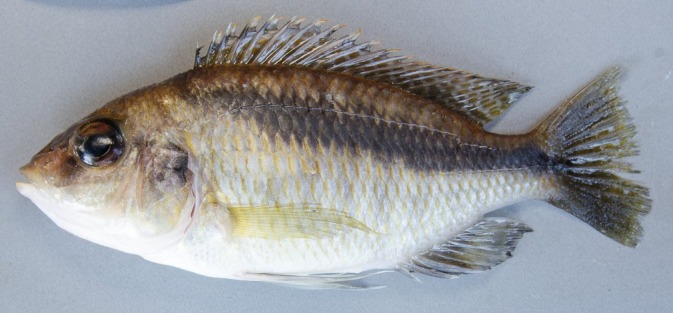
*Mylochromis durophagus*, holotype, UMZC 2016.18.13, 89.7 mm standard length (SL) male, freshly collected from Nkhata Bay on February 20, 2016. Photo H. Svardal.

**FIGURE 6:**
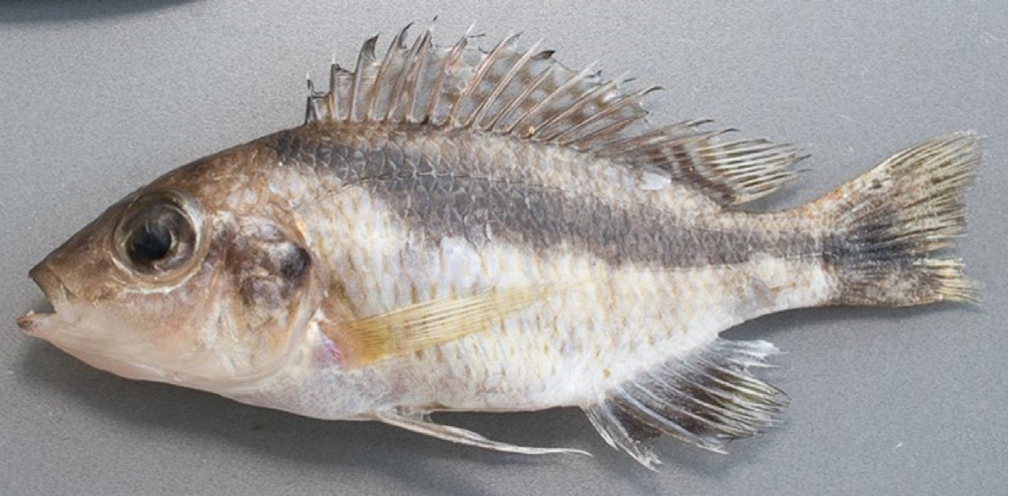
*Mylochromis durophagus*, paratype, UMZC 2016.25.2, 80.0 mm standard length (SL) female, freshly collected by scuba at Mphanga Rocks, Chilumba, February 23, 2016. Photo H. Svardal.

**FIGURE 7:**
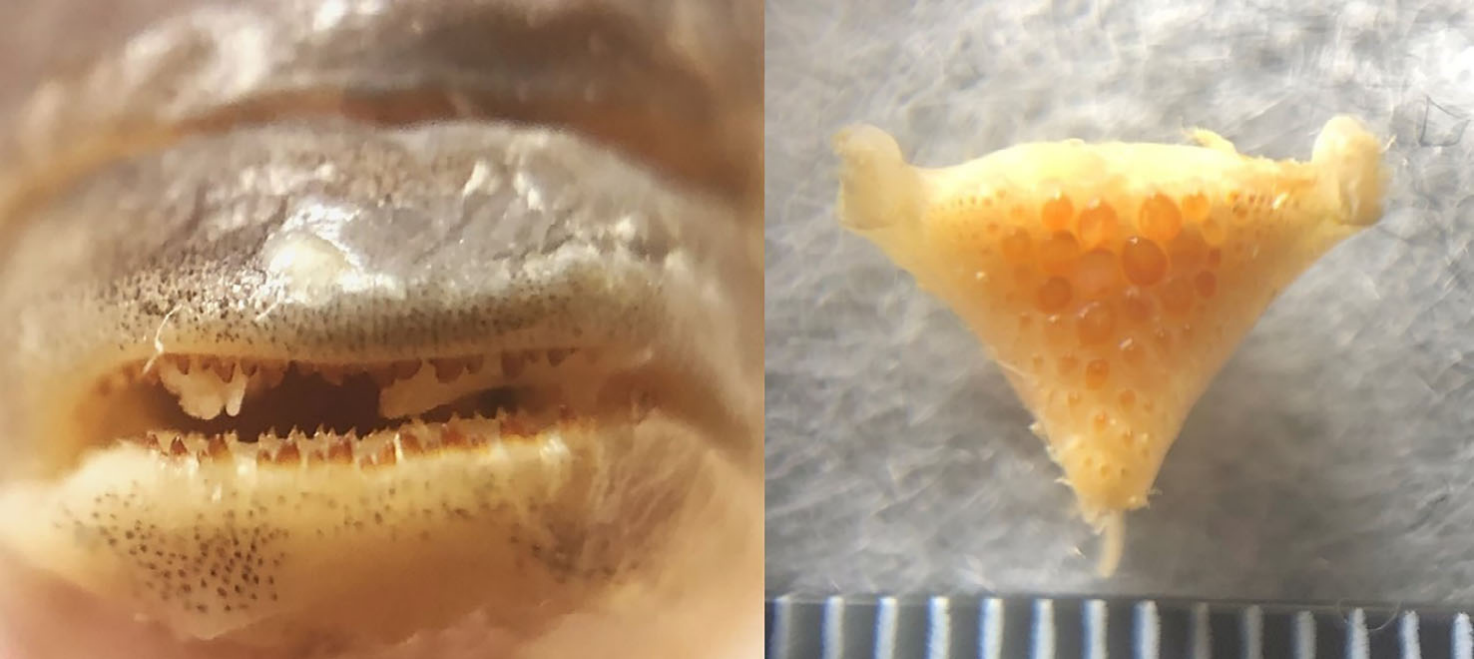
*Mylochromis durophagus*: jaw teeth of holotype (left) and lower pharyngeal bone of 80 mm paratype UMZC 2016.25.2.

**FIGURE 8:**
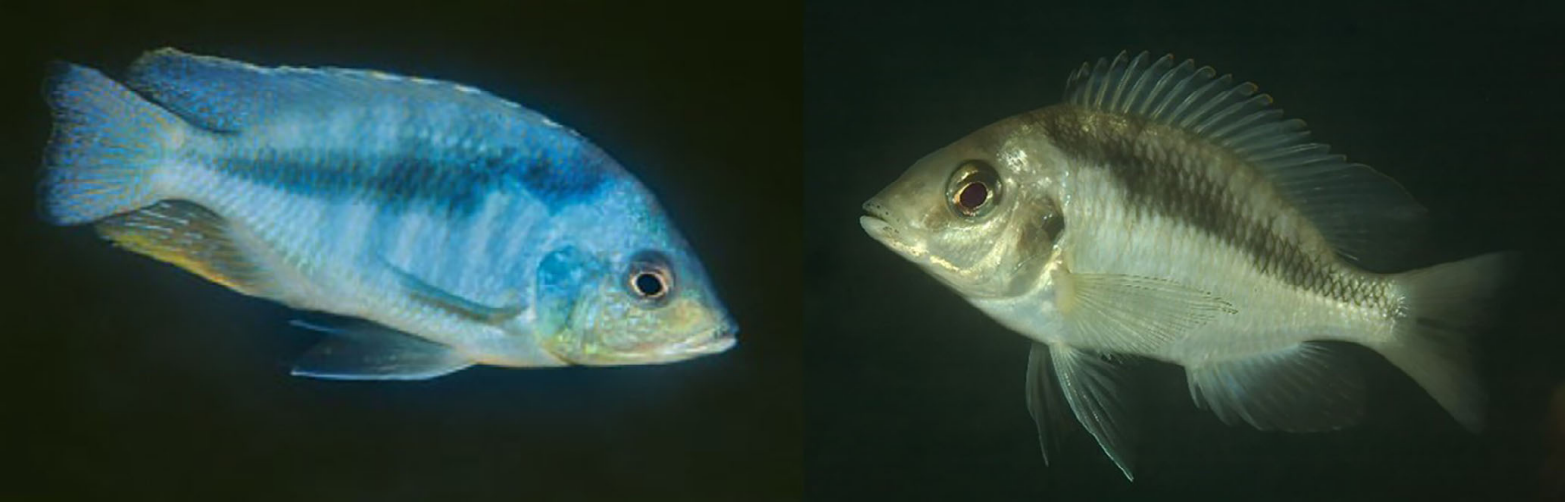
Live colouration of specimens provisionally identified as *Mylochromis* sp. “mollis chitande,” which may represent *Mylochromis durophagus*: male (left) and apparent female (right) from Maison Reef, Chilumba, northwest coast of Lake Malawi (right). Photos A. Konings.

Holotype: University Museum of Zoology, Cambridge: UMZC 2016.18.13, apparent male, 89.7 mm SL, collected by scuba at Nkhata Bay February 20, 2016, by Malawi Cichlid Genomic Diversity Survey (MCGDS) (Figures 5, 6, 7, 8).

Paratypes (2): UMZC 2016.18.15, apparent female, 67.5 mm SL, collected with holotype and one female UMZC 2016.25.2, 80.0 mm SL collected by scuba at Mphanga Rocks, Chilumba, February 23, 2016, by Malawi Cichlid Genomic Diversity Survey (MCGDS).

Etymology: “duro‐” = from durus, Latin, meaning “hard” + “‐phagus” = Latin, meaning “to eat,” referring to the presumed diet of hard‐shelled invertebrates indicated by the molariform pharyngeal dentition.

**FIGURE 9:**
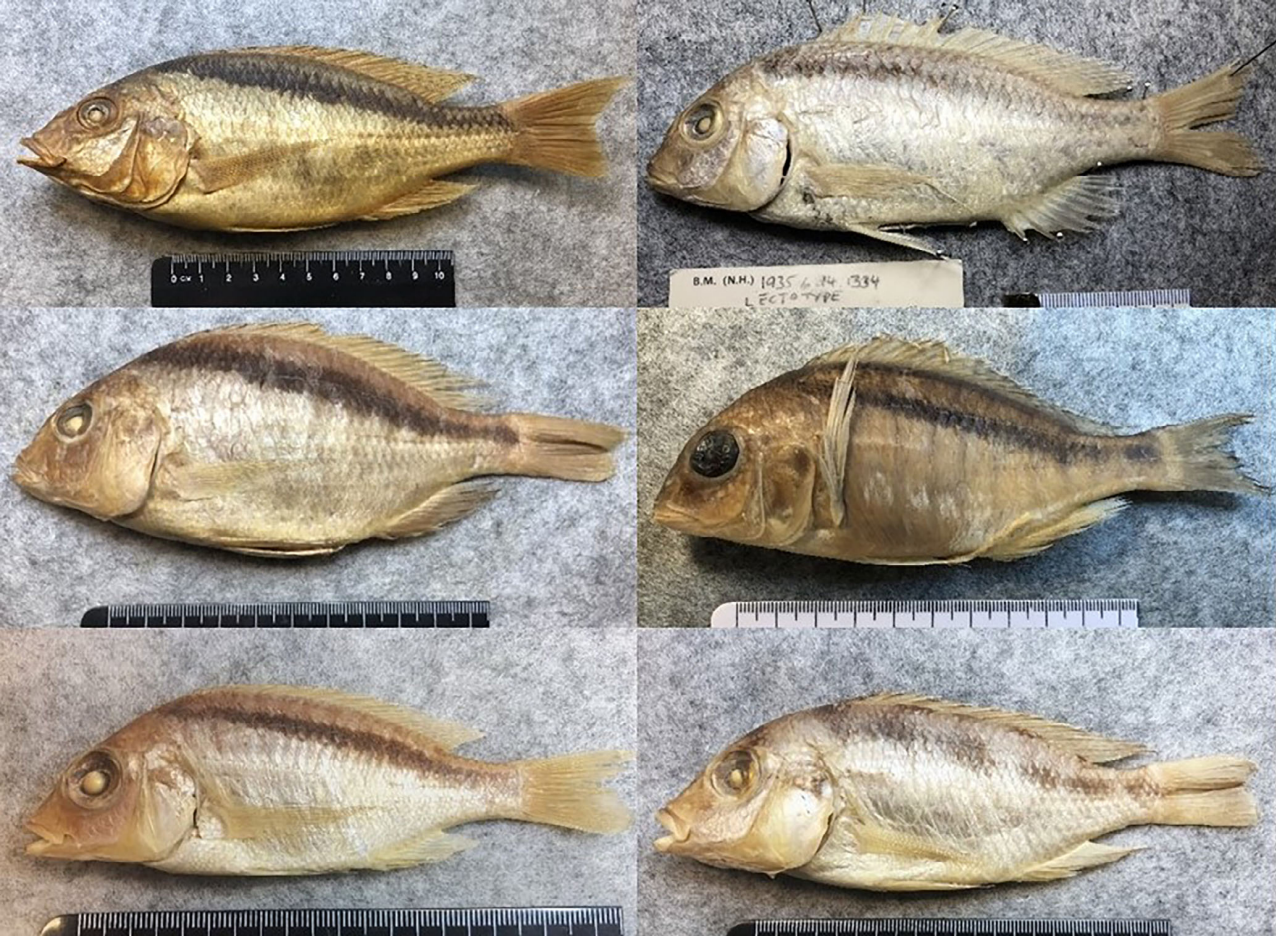
Comparable species: (top left) *Mylochromis melanonotus* lectotype BMNH 1921.9.6.163, 172 mm standard length (SL); (top right) *Mylochromis mollis* lectotype BMNH 1935.6.14.1334, 131.9 mm SL; (center left) *Mylochromis semipalatus* lectotype BMNH 1935.6.14.1321, 141 mm SL; (center right) *Mylochromis anaphyrmus*, BMNH 2024.6.25.1–4, 130.7 mm SL; (bottom left) *Mylochromis sphaerodon* lectotype 1921.9.6.147, 90 mm SL (bottom right) *Mylochromis mola*, BMNH1935.6.14.2357–2359, 122.4 mm SL. Photos Turner.

Diagnosis: the lower jaw dentition is “*Placidochromis*‐type,” with the outer series extending in a relatively straight line toward the posterior end of the jaw. This and the strong oblique stripe and lack of specialized morphology characterize the species as a member of the genus *Mylochromis*. The strongly molarized pharyngeal dentition distinguishes the species from all other known *Mylochromis*, except *M. anaphyrmus*, *M. mola*, and *M. sphaerodon* (Figure 9). *M. mola* differs by having a long snout (36.3%–40.3% vs. 33.4%–37.4% HL in *M. durophagus*), more acutely pointed head profile, more slender body (body depth 34.5%–37.1% vs. 39.1%–40.2% SL vs. in *M. durophagus*), and a blotchy, interrupted oblique stripe. *M. durophagus* has a much less steep head profile than *M. anaphyrmus* (40° vs. 56–67°) and a more upwardly angled gape (40° vs. 10–20°). *M. durophagus* also has a less steep head profile than *M. sphaerodon* (40° vs. 50–52°).

Description: body measurements and counts are presented in Table 2. *M. durophagus* is a medium‐sized (< 90 mm SL), laterally compressed (maximum body depth 2.35–2.42 times maximum width) cichlid fish with a rounded head profile, terminal mouth, and large eyes (29.3%–33.1% HL). Prominent, broad continuous oblique stripe on flanks, narrowing anteriorly (Figures 5 and 6).

**TABLE 2:** Morphometrics and meristics of *Mylochromis durophagus* sp. nov., holotype and two paratypes.

	Holotype	Paratypes	
Standard length (mm)	89.7	67.8	80
As % SL			
Body depth	40.2	39.7	39.1
Head length	35.8	35.7	36.9
Dorsal‐fin base Length	56.6	55.2	55.3
Anal‐fin base length	19.8	19.8	19.5
Predorsal length	37.9	39.4	39.9
Pre‐anal length	71.2	70.5	71.5
Prepelvic length	38.0	35.7	38.6
Preventral length	44.0	42.3	45.4
Caudal peduncle length	12.6	14.6	14.4
Caudal peduncle depth	13.5	13.0	13.3
As % head length			
Head width	46.7	45.9	45.1
Interorbital width	26.5	23.6	22.4
Snout length	37.4	33.5	36.6
Lower jaw length	35.2	36.4	34.2
Premaxillary pedicel length	30.5	31.0	28.5
Cheek depth	16.8	16.1	18.3
Eye diameter	29.3	33.1	31.9
Lachrymal depth	20.9	22.7	24.1
Ratios			
Maximum depth/head width	2.41	2.42	2.35
Caudal peduncle length/depth	0.93	1.13	1.08
Meristics			
Upper gill rakers	5	4	4
Lower gill rakers	10	10	11
Dorsal‐fin spines	17	17	17
Dorsal‐fin rays	11	10	10
Anal‐fin rays	9	9	9
Longitudinal‐line scales	33	32	32
Cheek scales	4	3	2

All specimens are moderately deep bodied and laterally compressed, with deepest part of body generally around first to fifth dorsal‐fin spine. Anterior upper lateral profile moderately convex, straight, angled about 40° to horizontal, from tip of snout to above eye, with little or no obvious bulge made by premaxillary pedicel, then curving gently to first dorsal spine. Jaws isognathous to slightly retrognathous, jaw teeth not prominent when mouth closed. Tip of snout well above level of upper insertion of pectoral fin and about level with the bottom of eye or slightly above. Lower profile curves from lower jaw tip to insertion of pelvic fins, then almost straight from pelvics to first anal spine. Mouth relatively small, gape angled about 40° to horizontal. Lips moderately fleshy in larger specimens, relatively thin in smallest. Posterior end of maxilla well in front of anterior margin of eye. Eye large, circular, and just below the head profile in lateral view. Lachrymal squarish with five openings.

Flank scales weakly ctenoid, with cteni becoming reduced dorsally, particularly anteriorly above upper lateral line, where they transition into a cycloid state. Scales on chest relatively large. Gradual transition in size from larger flank scales, as is typical in non‐mbuna Malawian endemic haplochromines (Eccles & Trewavas, 1989). Caudal fin densely scaled, over at least proximal three‐fourths.

Cephalic lateral‐line pores fairly inconspicuous, and flank lateral line shows usual cichlid pattern of separate upper and lower portions, with up to four pored scales after kink in upper lateral line and one to three smaller pored scales after line of flexion of hypurals.

Pectoral fins relatively long, to around vertical plane through third anal spine, whereas pelvics occasionally just reach first anal spine base. Filaments of dorsal and anal fins reaching just past base of caudal fin in larger specimens, but well short in smallest. Caudal fin emarginate.

Lower jaw bones relatively slight and lacking a mental process. Outer series of teeth in lower jaw stout, erect, prominent, and deeply implanted, bicuspid with pointed, obliquely truncated major cusps. Upper jaw outer series similar, but both cusps more rounded, larger becoming more pointed posteriorly. In both upper and lower jaws, inner teeth very small, scattered, not forming clear rows.

Lower pharyngeal bone heavily built with hemispherical molariform teeth central‐posteriorly. Gill rakers widely spaced, thick, blunt.

Female beige, paler on chest and belly, with a strong oblique stripe from nape to the caudal fin base, extending into fin rays. Dorsal fin has dull orange spots in two rows on spinous membranes, three oblique rows in soft dorsal; lappets dark in spinous portion. Caudal, anal, and pelvic fins dusky, pectorals yellowish. Mature male darker with golden spots on majority of flank scales. Dorsal, caudal, anal, and pelvic fins dark, dorsal and caudal with numerous orange spots. Dorsal‐fin lappets white with orange tips. Dark lachrymal stripe visible. Based on other Malawian haplochromines, this is unlikely to be full male breeding dress.

Distribution and ecology: known only from two specimens collected from rocky shores at Nkhata Bay and one at Mphanga Rocks near Chilumba in the northwestern part of Lake Malawi. All were collected in shallow water by scuba divers.

## 4 DISCUSSION

The two species described earlier are relatively poorly known and were not adequately distinguished during field collections, but only identified from preserved material examined as part of ongoing genomic studies (for some preliminary results, see Malinsky et al., 2018; Svardal et al., 2019; Turner et al., 2022). They were initially recorded as *Mylochromis cf. mollis*. Later investigation of the type material of *M. mollis* showed that this species is more slender in build, whereas the dissection of the pharyngeal bones indicated that we were likely dealing with two species, one with strongly molariform pharyngeal dentition and the other with papilliform dentition. Intraspecific polymorphism in pharyngeal structures is well known from a Mexican cichlid, *Herichthys minckleyi* (Bell et al., 2019). Intra‐population variation in pharyngeal dentition in African cichlids is relatively poorly known: some variation occurs in the Lake Malawi rocky shore species *Labidochromis caeruleus* Fryer 1956, but the relatively small sample size (Lewis, 1982) makes it difficult to know if this is a discrete polymorphism or continuous variation. There is also variation in pharyngeal bones within the Lake Masoko population of *A. calliptera*, although much of this is associated with divergence among ecotypes associated with microhabitat preferences (Carruthers et al., 2022; Malinsky et al., 2015). However, it generally appears that such clear differences in lower pharyngeal dentition correspond to species differences rather than polymorphisms (Eccles & Trewavas, 1989). Although the sample sizes in the present study are relatively small, the molariform and papilliform individuals also showed non‐overlapping differences in HL as %SL, premaxillary pedicel length as %HL and maximum body depth/head width (Tables 1 and 2), indicating that the two forms represented different morphotypes in unconnected traits consistent with them being different species. As with many other very similar Malawian cichlids, with practice, they could probably be differentiated by a subtle difference in overall appearance, in this case, largely from the head profile, without the need for dissection of the pharyngeal bones.

The two new species, described earlier, add to the long list of species within *Mylochromi*s. Many undescribed Malawi cichlids have been known via informal names since Ribbink et al. (1983) popularized the practice in their groundbreaking natural history overview of the rocky shore species of the Malawian coast and built on in works such as the successive books by Konings (1989–2016). Konings' most recent work (2016) lists 38 *Mylochromis* species, of which 17 are apparently undescribed. This is also the number listed on the “Cichlid Room Companion” website (cichlidae.com), which is regularly updated based on both academic and aquarium‐hobbyist literature.

It is not always straightforward to match formal descriptions with “field species” shown by Konings and other workers, whose identifications are based largely on underwater photographs. In most cases, details of morphological features such as dentition, gill rakers, and pharyngeal jaws are unknown. It is also unclear whether the field identifications of relatively rare species really correspond to the described species. This is attested to by the changing (improving!) names given to same taxon (sometimes the same photograph) across successive edition of Konings' book. Equally, a number of described species are known from a relatively small number of preserved specimens and may not all actually represent different species: *Mylochromis balteatus* and *Mylochromis melanotaenia* are only separated on the key given by Eccles and Trewavas (1989) on the basis of the number of teeth in the outer series of the lower jaw (51–53 vs. 38–46), although differences in lip thickness and position of lateral stripe are also mentioned in the text. *Mylochromis guentheri* and *M. mollis* appear to differ only in that the former has retrognathous jaws and procumbent lower jaw teeth (Eccles & Trewavas, 1989), but this is by no means obvious in all specimens in the type series.

Although voucher specimens and formal descriptions give a good insight into morphological traits, live colors may not be captured well. The specimens at the Cambridge Museum were initially collected by the Malawi Cichlid Genomic Diversity Survey (MCGDS) during 2016: a variety of sources and techniques were employed. The material of *M. rotundus* and *M. durophagus* were obtained by divers collecting fish on rocky shores, but these species were not individually observed underwater and were not photographed until postmortem.

Tentatively, it seems that the papilliform *M. rotundus* may be conspecific with Konings' *Mylochromis* sp. “mollis north” and the molarifom *M. durophagus* with Konings' *Mylochromis* sp. “mollis chitande.”

*Mylochromis* sp. “mollis north” was recorded from Hora Mhango, Kakusa, and Katale Island, all sites north of Ruarwe in the far northwest of the lake, whereas *M. rotundus* was only collected from the Chilumba area, the more southerly sites of Konings were not sampled in the 2016 collecting trip. Konings reports the species as feeding from sand. Males are reported to have a yellow breast and to dig a “cave‐crater” bower.

Konings (2016) records *Mylochromis* sp. “mollis chitande” from a number of sites around the Chilumba area (Mdoka to Maison Reef), which overlaps with the range of the specimens of *M. durophagus* (Mphanga Rocks, Nkhata Bay). Konings recorded the species as uncommon, and mainly inhabiting the rock‐sand interface area (“intermediate habitat”), picking invertebrates mainly from rocky substrates. He comments on two of the main identification features of *M. durophagus*, the pointed snout and broad, irregular oblique stripe. Underwater photographs show a similar stripe pattern to *M. durophagus*, in particular, the way the stripe is narrow anteriorly and then suddenly becomes much wider below around the sixth to eighth dorsal‐fin spine. There is also a hint to a large dark blotch on the operculum in the illustrations (Figure 8) similar to that seen in the type specimens (Figures 5 and 6).
